# QML-AiNet: An immune network approach to learning qualitative differential equation models

**DOI:** 10.1016/j.asoc.2014.11.008

**Published:** 2015-02

**Authors:** Wei Pang, George M. Coghill

**Affiliations:** School of Natural and Computing Sciences, University of Aberdeen, Aberdeen AB24 3UE, UK

**Keywords:** Qualitative model learning, Artificial immune systems, Immune network approach, Compartmental models, Qualitative reasoning, Qualitative differential equation

## Abstract

In this paper, we explore the application of Opt-AiNet, an immune network approach for search and optimisation problems, to learning qualitative models in the form of qualitative differential equations. The Opt-AiNet algorithm is adapted to qualitative model learning problems, resulting in the proposed system QML-AiNet. The potential of QML-AiNet to address the scalability and multimodal search space issues of qualitative model learning has been investigated. More importantly, to further improve the efficiency of QML-AiNet, we also modify the mutation operator according to the features of discrete qualitative model space. Experimental results show that the performance of QML-AiNet is comparable to QML-CLONALG, a QML system using the clonal selection algorithm (CLONALG). More importantly, QML-AiNet with the modified mutation operator can significantly improve the scalability of QML and is much more efficient than QML-CLONALG.

## Highlights

• We propose an immune network approach to learning qualitative models.
• The immune network approach improves the scalability of learning.
• The mutation operator is modified for searching discrete model space.
• Promising results are obtained when learning compartmental models.

## Graphical abstract

**Figure:**
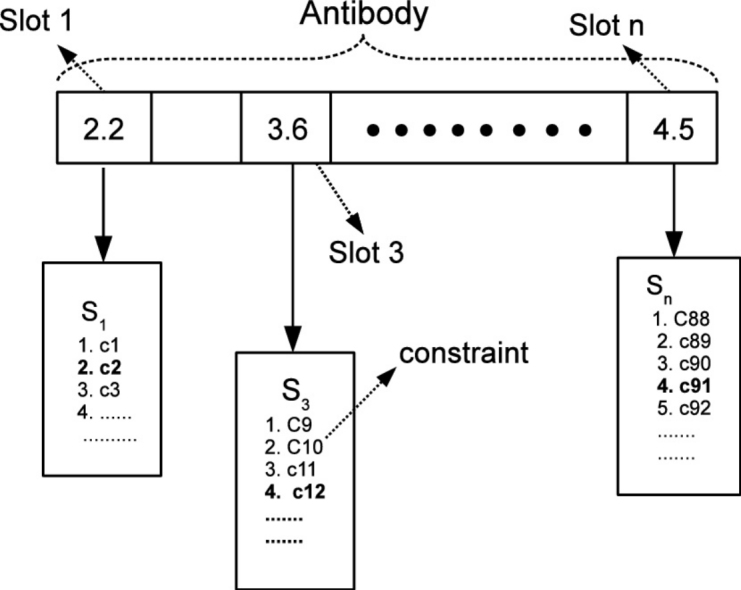

## 1 Introduction

Qualitative Reasoning (QR) [1] is a field devoted to reasoning about complex systems at a qualitative level when only imprecise data and incomplete knowledge are available. In QR research there exist a few subfields, for instance, qualitative simulation (QS) based on qualitative differential equations (QDEs) [2], [3], [4], qualitative process theory (QPT) [5], [6], QDE model learning (QML, and see [7] for a review), qualitative tree induction [8], [9], and most recently, learning qualitative models by estimating partial derivatives [10] and learning QPT models [11].

Among the above mentioned subfields of QR, QDE model learning (QML) continuously receives some attention in the last two decades, because it has promising application potential in systems identification [12] in contexts where data may be sparse and noisy. Both QML and QS use the formalism of QDE, which was initially used in QSIM [13], and later extended in other QS systems, such as FUSIM [3] and *Morven* [4]. QS starts from a QDE model for a complex system, and derives possible behaviours from this QDE model. QS assumes that a QDE model can be obtained from either domain knowledge or experts, while QML considers the situation that a QDE model for a dynamic system cannot be straightforwardly obtained, and aims to automatically infer such QDE model from available data and knowledge.

Over the last two decades a few QML systems have been developed to solve different problems, and examples of these systems include GOLEM [14], GENMODEL [15], [16], MISQ [17], [18], [19], QSI [20], QME [21], ILP-QSI [22], and the most recent system QML-Morven [7], [23].

However, there exist two issues in QML research, which have been little studied: first, the scalability of QML: that is, a QML algorithm may be inefficient, or even intractable when dealing with large-scale search spaces, resulting from the complexity of problems and/or the incomplete knowledge, for instance, the presence of hidden variables (those variables that cannot be observed in experiments). Second, the highly multimodal nature of QML search spaces. In the presence of incomplete data and knowledge, it is often found that there exist many local optima in QML search spaces. This means an inappropriate search strategy will make a QML system get trapped in local optima easily.

Heuristic algorithms could be employed as search strategies to QML to improve the learning performance. In this regard several attempts have been made in previous research, for instance, the branch-and-bound search used in ILP-QSI, the genetic algorithm used in QME, and the backtracking with forward checking algorithm employed in our previous work [24], [23]. However, all these heuristic algorithms cannot well address both of the above-mentioned two issues in QML at the same time: backtracking and branch-and-bound search may have the scalability issue when searching large-scale model spaces; genetic algorithms could scale well, however, they tend to converge to a single solution and cannot deal with multimodal search spaces. Many other heuristic algorithms which have not been applied to QML, including hill climbing, A*, and simulated annealing, also suffer from either the scalability issue or the ineffective search in multimodal search spaces.

Considering the above facts, we proposed immune-inspired approaches to QML as they have been proven to be effective in solving many problems with large-scale and multi-model search spaces. In previous work we first proposed a pilot system EQML [25], [26], an evolutionary qualitative model learning framework. In EQML, CLONALG [27], [28], an evolutionary and immune-inspired algorithm based on the clonal selection theory [29] in immunology, was adapted to QML and its performance was compared against a genetic algorithm. Experimental results obtained from EQML showed that immune inspired approaches were feasible, and had great potential to be applied to QML. The CLONALG algorithm for QML was later improved in [24], and the resulting QML system is termed QML-CLONALG in this paper. Experiments with QML-CLONALG demonstrated that immune inspired approaches were suitable for highly multi-modal search spaces of QML, and the scalability of QML could be improved when dealing with large-scale search spaces.

The success of QML-CLONALG is due to the nature of CLONALG, which maintains a diverse antibody population through clonal, hypermutation, and selection operations performed on antibodies. This motivates us to continue the study of other immune inspired approaches to QML. In particular, from the literature of Artificial Immune Systems (AIS), we know that Opt-AiNet [30], [31], an immune network approach to optimisation problems, can more effectively deal with large-scale and multimodal search spaces compared to CLONALG. This is because apart from using the clonal selection mechanism, OptAiNet also introduced interactions between antibodies, that is, the antibody population will undergo a network suppression procedure, and those antibodies with lower fitness values among similar antibodies will be eliminated to make the search diverse. Furthermore, the behaviour of the intrinsic diversity mechanisms of Opt-AiNet has been experimentally studied and confirmed [32].

Based on the above considerations, in this paper we will investigate the application of Opt-AiNet to better address the issues of scalability and highly multimodal search spaces in QML. We proposed an immune network approach with modified mutation operator, which we termed QML-AiNet (MM), to qualitative model learning. Compared to existing QML systems, the advantages of our system is as follows:

• Compared to QML systems using deterministic search algorithms, such as backtracking and branch-and-bound search, the proposed algorithm is more scalable to large search spaces.
• Compared to QME, which uses a genetic algorithm as its model learning strategy, the proposed algorithm can better deal with search spaces with multimodal fitness landscapes.
• More importably, compared to previous immune-inspired QML systems, our algorithm is more scalable to extremely large search spaces. Experiments have shown that the proposed QML-AiNet (MM) is two to three orders of magnitude more efficient than our previous immune-inspired systems QML-CLONALG [24] and QML-AiNet (OO) [33], an earlier version of QML-AiNet using the original mutation operator.

The rest of this paper is organised as follows: in Section 2 we first introduce some basic concepts in qualitative reasoning. This is followed by Section 3, in which we give a brief description of *Morven*, a qualitative reasoning framework used in this research to represent and verify models. In Section 4, we give a formal description to explain that QML is a search and optimisation problem. The main work of this research, QML-AiNet, is described in detail in Section 5. Then in Section 6 we report a series of experiments to evaluate the performance of QML-AiNet. Finally, in Section 7 we draw the conclusion and explore some future work.

## 2 Basic concepts in qualitative reasoning

### 2.1 Qualitative differential equations

A qualitative differential equation (QDE) is formally defined as a tuple <*V*, *Q*, *C*, *T*> [2], where *V* represents the set of *qualitative variables*; *Q* is the set of *quantity spaces*, each of which is associated with a qualitative variable in *V*; *C* is a set of *qualitative constraints* that apply to the variables in *V*; *T* is a set of transitions between *qualitative states*. Simply speaking, a QDE is the conjunction of all its qualitative constraints, which link the qualitative variables and express the relations among these variables. As for the set of quantity spaces *Q*, different qualitative reasoning engines may have different forms of representation, but all qualitative variables are restricted to only take qualitative values from their associated quantity spaces. In *Morven* [4], [34] (and the early system FuSim [3]), a quantity space is composed of several trapezoidal fuzzy numbers, each of which is represented by the fuzzy 4-tuple parametric representation: <*a*, *b*, *α*, *β*> (details of which can be found in [3]).

The set of qualitative constraints C is composed of two types of constraints: *algebraic constraints* and *functional constraints*. The former represent algebraic relations between variables as in quantitative mathematics, for instance, *addition*, *subtraction*, and *multiplication*; the latter describes incomplete knowledge between two variables, for example, the monotonically increasing and decreasing relations, which state that one variable will monotonically increase with the increase/decrease of another. The *function* constraints in FuSim and *Morven* are such functional constraints, and they define many-to-many mappings which allow flexible empirical descriptions between two variables without knowing the exact mathematical relation.

Table 1 lists some *Morven* constraints and their corresponding mathematical equations. In this table variables in the right column such as *X*(*t*) are continuous functions of time *t*. *f* is a function that is continuously differentiable over its domain. In the constraints listed in the left column of the table, the label *dt* means *derivative*, and the integer immediately following it indicates which derivative of the variable (0 means the magnitude). This means each place in a *Morven* constraint can represent not only the magnitude, but also arbitrary derivative of a variable.

**Table 1:** Some qualitative constraints in *Morven* and their corresponding mathematical equations.

*Morven* constraints	Mathematical equations
sub (dt 0 Z, dt 0 X, dt 0 Y)	*Z*(*t*) = *X*(*t*) − *Y*(*t*)
mul (dt 0 X, dt 0 Y, dt 0 Z)	*Z*(*t*) = *Y*(*t*) * *X*(*t*)
Function (dt 0 Y, dt 0 X)	*Y*(*t*) = *f*(*X*(*t*))
sub (dt 1 Z, dt 0 X, dt 0 Y)	*dZ*(*t*)/*dt* = *X*(*t*) − *Y*(*t*)
Function (dt 1 Y, dt 0 X)	*dY*(*t*)/*dt* = *f*(*X*(*t*))

From the above introduction to QDEs we see that a QDE is an abstraction of a set of ordinary differential equations (ODEs) [35] in the sense that the functional relations of a QDE correspond to an infinite number of quantitative mathematical functions, and the qualitative values assigned to variables in a QDE represent various quantitative values.

### 2.2 Qualitative simulation based on QDE

Qualitative simulation concerns solving a QDE and thus predicting the possible behaviours of a system. A QDE simulator, such as *Morven*, can take as input a QDE model and generate all possible *qualitative states* and possible transitions between these states. A qualitative state is a complete assignment of qualitative values to all qualitative variables of the system, and it describes a “snapshot” of the system qualitatively. The possible transitions between states are defined by transition rules, the definition of which is not given here as it is beyond the scope of this paper. A *qualitative behaviour* is a series of qualitative states linked by their legal transitions.

## 3 The *Morven* framework and JMorven

### 3.1 The Morven framework

In this research we use *Morven* [4] to represent QDE models. In *Morven*, qualitative constraints are distributed over multiple *differential planes*. The *0th* differential plane contains the constraints, which can represent a model used for numerical simulation. The constraints in a higher differential plane are obtained by differentiating the corresponding constraints in the preceding differential plane.

Qualitative variables in *Morven* are in the form of variable length vectors. The first element in the vector is the magnitude of the variable, the *i*th (*i* > 1) element is the (*i* − 1)th derivative. One can include as many derivatives as necessary.

As mentioned in Section 2.1, qualitative values in *Morven* are in the form of a fuzzy four-tuple <*a*, *b*, *α*, *β*>. However, as in this research the fuzzy mechanism is not used, each qualitative value in *Morven* will degenerate into an interval value (*a*, *b*), where *a* and *b* are real numbers or −∞ and +∞, denoting the lower and upper bounds of the qualitative value, respectively (*a* ≤ *b*). Accordingly the interval arithmetic operations will be used to calculate values based on constraints.

**Fig. 1:**
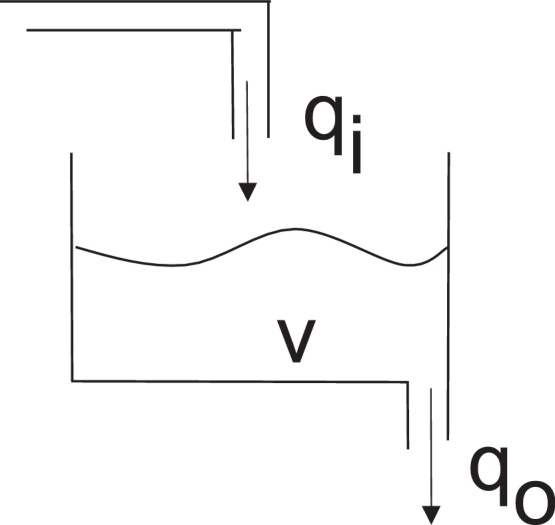
The single tank system.

The single tank system shown in Fig. 1 is used as an example to demonstrate how *Morven* is used to represent QDE models. The quantitative model for a linear version of this system is as follows:

$$q_{o}=k*V$$

$$\frac{\mathrm{dV}}{\mathrm{dt}}=q_{i}-q_{o}$$

where *V* is the volume of the liquid in the tank, *q*_*i*_ is the inflow, *q*_*o*_ is the outflow, and *k* is a positive constant coefficient determined by the cross sectional area of the tank and the density of the liquid.

The corresponding *Morven* model is shown in Table 2. This model is composed of four constraints, *C1* to *C4*, which are distributed over two *differential planes*. The meaning of these constraints has been explained in Section 2.1, and the corresponding quantitative relation for each constraint is shown on the right hand side in the brackets. For variable *V*, the magnitude, the first and second derivatives are used; for variable *q*_*o*_ and *q*_*i*_, only the magnitude and the first derivative are used.

**Table 2:** The *Morven* model for the single tank system.

Differential Plane 0	
*C1*: Function (dt 0 *q*_*o*_, dt 0 V)	(*q*_*o*_ = *k* * *V*)
*C2*: sub (dt 1 V, dt 0 *q*_*i*_, dt 0 *q*_*o*_)	(*V*′ = *q*_*i*_ − *q*_*o*_)

Differential Plane 1	
*C3*: Function (dt 1 *q*_*o*_, dt1 V)	($q_{o}^{\prime }=k*V^{\prime }$)
*C4*: sub (dt 2 V, dt1 *q*_*i*_, dt1 *q*_*o*_)	($V^{\prime \prime }=q_{i}^{\prime }-q_{o}^{\prime }$)

If all the qualitative variables (including their magnitudes and derivatives) use the signs quantity space, which is shown in Table 3, the mappings of the *Function* in constraint *C1* and *C3* are given in Table 4, in which “1” stands for the existence of a mapping between variables A and B.

**Table 3:** The signs quantity space.

Quantity	Range
negative (−)	(−∞, 0)
zero (0)	(0,0)
positive (+)	(0, ∞)

**Table 4:** Function mappings using the signs quantity space.

Function (*A*, *B*)	negative	zero	positive
negative	1	0	0
zero	0	1	0
positive	0	0	1

### 3.2 JMorven

JMorven [34], [36] is a Java implementation of *Morven*, and it will be used as a model verification component in this research. Candidate models generated by QML-AiNet, the proposed algorithm, will be simulated by JMorven and the output will be compared against given data.

**Fig. 2:**
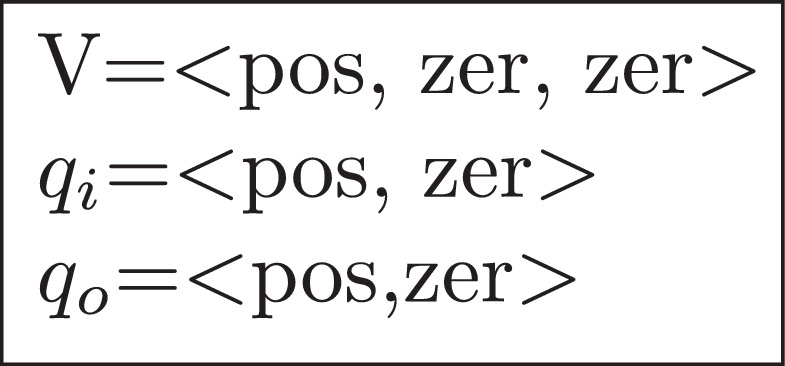
A qualitative state of the single tank in *Morven*.

The output of JMorven for a QDE model could be either an *envisionment* containing all possible qualitative states and their legal transitions, or a behaviour tree which is part of the envisionment. As mentioned in Section 2.2, a qualitative state is a complete assignment of qualitative values to all qualitative variables of the system, and one possible qualitative state of the single tank system described by *Morven* is shown in Fig. 2. In this figure the assignment *V* =< *pos*, *zer*, *zer* > means that the magnitude of V is *positive*, the first and second derivatives are *zero* (all values are taken from the signs quantity space defined in Table 3). It is similar for the assignments of *q*_*i*_ and *q*_*o*_.

## 4 QDE Model Learning: a search and optimisation problem

In this section we give a formal description of QDE model learning (QML). QML is considered as the inverse of qualitative simulation (QS), which is described in Section 2.2. QML aims to infer possible QDE models based on available knowledge and data, which could be either quantitative and qualitative. A Q2Q (Quantitative-to-Qualitative) conversion algorithm [22] is used if the given data are quantitative.

For a specific problem *P*, given the background knowledge *BK*, the set of all variables *V* (which may also include hidden variables), and the set *R* that contains all possible qualitative relations of these variables (such as monotonically increasing or decreasing relations, and algebraic relations), we can generate a set *CS* containing all possible constraints by using all combinations of elements in *R* and *V*, which is shown as follows:

(1) CS={c=(r,a,b,d)|r∈R,a,b∈V,d∈V∪{∅}}.

In the above a qualitative constraint is represented by a four-tuple vector *c*, where *r* denotes a qualitative relation, and *a*, *b*, *d* are variables. In addition, if *r* is a functional relation, *d* will be empty. For example, in *Morven*, constraint *Sub(dt 0 Z, dt 0 X, dt 0 Y)* is represented as *c* = (*Sub*, *Z*, *X*, *Y*), and *Function (dt 1 X, dt 0 Y)* is represented as *c* = (*Function*, *X*′, *Y*). In this sense *V* in Eq. (1) could also include derivatives of variables if *Morven* is used.

If we denote *GDS* as the *given data set* and consider the background knowledge *BK*, we can use *GDS* and *BK* to filter out the inconsistent constraints in *CS*, and generate a filtered constraint set *FCS*:

(2) FCS={c|c∈CS,   c   s.t.   BK,   c   s.t.   GDS}.

The meaning of the above formula is as follows: each constraint in *FCS* is consistent with *BK* and covers *GDS*. The implicit QDE model space *QMS* contains all possible QDE models generated from *FCS*, as shown below:

(3) QMS={m|m∈℘(FCS)}.

In the above *m* is a possible QDE model and the symbol ℘ stands for the power set operation, which means *m* could be the conjunction of constraints from any subset of *FCS*. So the size of this implicit search space is

(4) $$|\mathrm{QMS}|=2^{\left|\mathrm{FCS}\right|},$$

where the symbol “| · |” denotes the cardinality of a set, or the number of elements in a set. The task of QML is to find a candidate set of models, denoted as *CM*, and each element (a model) *m* of *CM* satisfies *BK* and covers *GDS*, as written below:

(5) $$\begin{array}{ccc} & & {\mathrm{QML}}_{P}(\mathrm{QMS})=\mathrm{CM}=\{m|m\in \mathrm{QMS},\\ & & m\;s.t.\;\mathrm{BK},\;\mathrm{QS}(m)⊇\mathrm{GDS}\}. \end{array}$$

In the above *QML*_*P*_ stands for QDE model learning for Problem *P*; *QS*(*m*) stands for the qualitative simulation of Model *m*, and the result is a set containing all qualitative states obtained from the simulation. From Eqs. (3), (5) we see that QML is essentially a search and optimisation problem, that is, to search for the best models from *QMS* that satisfy Eq. (5). Note that due to the complexity of the problem and the presence of incomplete knowledge and data, the size of the search space *QMS* could be too large to feasibly enumerate all its elements. In the search process it is often the case that only a portion of the search space is explored by appropriate search strategies for large-scale search spaces.

## 5 QML-AiNet

We propose QML-AiNet, a novel QML system which employs an Opt-AiNet [30], [31] based search strategy. Apart from the core search strategy, the other components of QML-AiNet are largely based on QML-CLONALG [24]. Like QML-CLONALG, QML-AiNet uses *Morven* (described in Section 3.1) to represent models, and JMorven (described in Section 3.2) to verify models. As in QML-CLONALG, QML-AiNet made use of well-posed model constraints [22] proposed in ILP-QSI, which serves as background knowledge (*BK*) to narrow the search space.

In the rest of this section, we first introduce the pre-processing phase of QML-AiNet, then describe in detail the core algorithm of QML-AiNet. In particular, we discuss two mutation strategies used in QML-AiNet.

### 5.1 Pre-processing phase

As in QML-CLONALG, the pre-processing phase of QML-AiNet includes four sub-components: *Constraint generation*, *Constraint filtering*, *Calculation of conflict set and dependency set*, and *Constraint set partition*. These four components will be briefly introduced in this section, and for more details, the reader is directed to [24].

  (1) *Constraint generation*: generate all possible constraints to construct *CS*, as defined in Eq. (1).

  (2) *Constraint filtering*: generate the filtered constraint set *FCS*, as defined in Eq. (2).

  (3) *Pre-calculation*: For each constraint in *FCS*, calculate its *conflict set* and *dependency set*. The conflict set for a constraint *c* contains all constraints in *FCS* that conflict with *c*. Two constraints are conflicting if they are logically inconsistent or redundant if they appear in the same QDE model.

The dependency set for a constraint *c* contains all constraints in *FCS* that *depend on c*. We say that constraint *c1* depends on constraint *c2* if the leftmost variable of *c2* appears in any non-leftmost position of *c1*. The calculation of the dependency set is used for checking the causal ordering [37] of a model.

The pre-calculation is for the ease of future computation, and the results are stored for later use.

  (4) *Constraint set partition*: A *defining constraint* for a variable *v* is the constraint in which *v* is the leftmost variable. *FCS* is divided into several subsets *S*_*i*_ (*i*=1 to *N*, and *N* is the number of variables including hidden variables), and each of these subsets contains all defining constraints for the same variable, say variable v, as shown below:

(6) $$\begin{array}{ccc} S_{i}=\{c|c=(r,v,b,d),c\in \mathrm{FCS},\;r\in R,b\in V,d\in V\cup \{\emptyset \}\}. & & \end{array}$$

where *R* and *V* have the same definitions as in Eq. (1).

We define the following set:

(7) $$\mathrm{DS}=\{S_{1},S_{2},\ldots ,S_{N}\},$$

where each element *S*_*i*_ (*i* ∈ [1, *N*]) in *DS* is the one defined in Eq. (6), and *N* is the number of variables in the model. We then give the following theorem of reduced search space under well-posed model constraints:

Theorem 1 *(The theorem of reduced search space)*[24]

*A qualitative model used in Morven satisfying the well-posed model constraints*[22]*must include one and only one defining constraint for each of the system variables with either 0th or first derivative in the 0th differential plane.*

The proof of the above theorem is given in [24]. In this theorem, *system variables*, also called *non-exogenous variables*, are understood as those variables which are not determined from outside of the system. For instance, in the single tank system shown in Fig. 1, variables *V* and *q*_*o*_ are system variables, while *q*_*i*_ is an exogenous variable. The well-posed model constraints are a set of reasonable constraints which a correct QDE model should obey, and these constraints include: (a) *Size*: the model size is defined as the number of constraints in the 0th differential plane of the model. The model size must be within the given range. (b) *Completeness*: the model must include all known variables. (c) *Logical consistency*: there are no conflicting or redundant constraints in the model. (d) *Dimensional consistency*: each variable has the same dimension in all constraints in which it appears. (e) *Language*: a model has to satisfy the given language constraints, such as the number of instances of any qualitative relation in the model must be below some pre-specified limit. Detailed discussion is given by Camacho [38]. (f) *Connection*: all system variables should appear in at least two constraints. (g) *Singularity*: there are no disjoint sub-models. (h) *Causal ordering*: the model can be causally ordered, as described in [37]. (i) *Coverage*: the model can cover all the given data.

According to the theorem of reduced search space, in Eq. (7) for each *S*_*i*_ in *DS*, if *S*_*i*_ is a set of defining constraints for a non-hidden variable, a well-posed model must include one and only one constraint taken from this *S*_*i*_; if *S*_*i*_ is a set of defining constraints for a hidden variable, a well-posed model can include *at most* one constraint taken from *S*_*i*_. Based on the above description, the search space *QMS* in Eq. (3) can be significantly narrowed down:

(8) $$\begin{array}{ccc} & & {\mathrm{QMS}}_{\mathrm{wp}}=\{m|m=(c_{1},c_{2},\ldots ,c_{\left|\mathrm{DS}\right|}),\;c_{i}\in S_{i}\cup \{\phi \},\\ & & S_{i}\in \mathrm{DS},\;i=1,2,\ldots ,|\mathrm{DS}|\}. \end{array}$$

In the above *QMS*_*wp*_ stands for the qualitative model space under well-posed model constraints; *m* is a possible QDE model composed of several constraints *c*_*i*_; *ϕ* stands for an empty constraint; and |*DS*| is the number of elements in *DS*. From Eq. (8) we see that the size of the search space becomes

(9) $$|{\mathrm{QMS}}_{\mathrm{wp}}|=\prod_{i=1}^{\left|\mathrm{DS}\right|}T_{i},$$

where *T*_*i*_ is defined as

(10) $$T_{i}=\left\{\begin{array}{cc} |S_{i}|+1 & \;\forall c\in S_{i},c=(r,v,a,b),v\;\;\text{is a hidden variable}\\ \left|S_{i}\right| & \;\mathrm{Otherwise} \end{array}\right)$$

In the above |*S*_*i*_| is the number of constraints in *S*_*i*_. Eq. (9) indicates that even though the search space can be significantly narrowed down compared to the size of the implicit search space |*QMS*| shown in Eq. (4), the size of the search space still increases exponentially with the number of variables in the model.

### 5.2 Antibody encoding and decoding

The original Opt-AiNet for function optimisation employs the real number encoding. Each variable in the function is assigned a value within its range. In QML-CLONALG, the integer encoding is used: the antibody is composed of several slots, each of which corresponds to an element *S*_*i*_ (defined in Eq. (6)) in *DS* (defined in Eq. (7)). The integer assigned to each slot indicates the selection of a constraint in *S*_*i*_.

Similar to QML-CLONALG, in QML-AiNet an antibody is also composed of several slots, and each of them corresponds to a constraint subset *S*_*i*_ in *DS*. Unlike QML-CLONALG, in QML-AiNet the value assigned to each slot is a real number, which is the same as in the original Opt-AiNet. This is represented as follows:

(11) $$\mathrm{Ab}=\{{\mathrm{Sl}}_{1},{\mathrm{Sl}}_{2},\ldots ,{\mathrm{Sl}}_{\left|\mathrm{DS}\right|}\}.$$

In the above *Ab* stands for an antibody; *Sl*_*i*_ (*i* ∈ [1, |*DS*|]) represents the value assigned to the corresponding slot of *Ab*, satisfying ${\mathrm{Sl}}_{i}\in \mathbb{R}$ and 1 ≤ *Sl*_*i*_ ≤ |*S*_*i*_|.

The real number encoding is compatible with the affinity proportion mutation operator in QML-AiNet, which will be described in Section 5.4. As the real number encoding strategy is used, when we decode an antibody, each value *Sl*_*i*_ will be rounded off to its nearest integer, denoted as [*Sl*_*i*_]. If *Sl*_*i*_ is in the middle of two integers, the smaller integer will be taken. Then the newly obtained integer for each slot will be used as an index to retrieve the corresponding qualitative constraint in each *S*_*i*_. So after the decoding of an antibody represented by Eq. (11), the following model *m* will be obtained:

(12) $$m=\{c_{\left[{\mathrm{Sl}}_{1}\right]},c_{\left[{\mathrm{Sl}}_{2}\right]},\ldots ,c_{\left[{\mathrm{Sl}}_{\left|\mathrm{DS}\right|}\right]}\}.$$

In the above $c_{\left[{\mathrm{Sl}}_{i}\right]}$ means the [*Sl*_*i*_]-th constraint in *S*_*i*_.

**Fig. 3:**
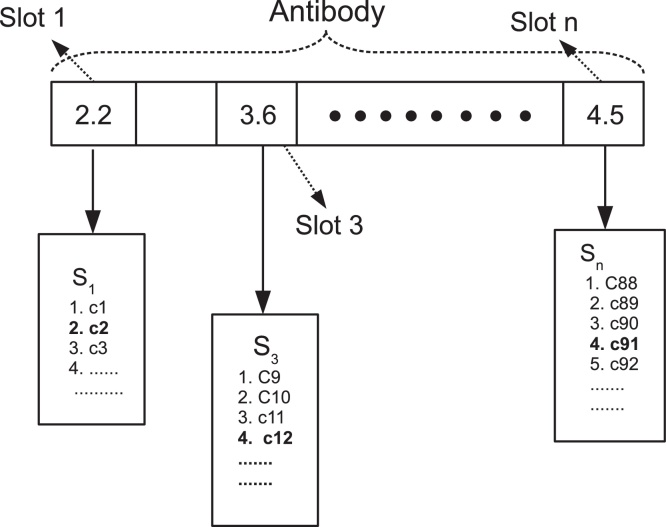
The antibody encoding and decoding of QML-AiNet.

Fig. 3 shows an example of the antibody encoding and decoding in QML-AiNet. In this figure, the antibody has *n*=|*DS*| slots, which correspond to *S*_1_, *S*_2_, … *S*_*n*_. In Slot 1 the current value is 2.2. After decoding we get an integer 2, so the second constraint *c*2 in *S*_1_ is selected (indicated in bold font). In Slot *n* the assigned value is 4.5, which is in the middle of 4 and 5. After decoding we obtained integer 4, and the fourth constraint *c*91 in *S*_*n*_ is selected. It is similar for the other slots. At the end of this process the model decoded from the antibody shown in Fig. 3 contains constraints *c*2, *c*12, and *c*91.

### 5.3 Fitness evaluation

The fitness evaluation is based on the well-posed model constraints, and takes the same scoring system as in QML-CLONALG. We note here that in QML-CLONALG this process is called the affinity evaluation, and in QML-AiNet the affinity has a different meaning which will be defined later in Section 5.5.

In the fitness evaluation process an antibody is first decoded to a model, then this model is checked against the well-posed model constraints described in Section 5.1. In the fitness evaluation the most expensive procedure is the coverage test of a model, for which qualitative simulation has to be used.

### 5.4 Mutation

In this research we first followed the original mutation method of Opt-AiNet, that is, for each slot of the antibody, the current value *C* will be mutated to a new value *C*′ according to the following equations:

(13) $$C^{\prime }=C+\alpha \times N(0,1)$$

(14) $$\alpha =\frac{1}{\beta }\times e^{-f^{*}}$$

In the above equations, f^*^ is the normalised fitness with the range [0,1]. *N*(0, 1) is a Gaussian random variable which has a mean value of 0 and standard deviation of 1. $e^{-f^{*}}$ is the inverse exponential function. *α* stands for the amount of mutation. *β* is a parameter that adjusts this exponential function. Because we expect the mutated value is different after decoding, in all experiments the value of *β* is set to 1, instead of the default value 100 in Opt-AiNet.

However, even if the value of *β* is set very low, in the experiments we found that the mutation is still not efficient enough to explore the model space. This is because the above mutation operator was developed for continuous function optimisation, and it does not consider the discrete nature of the qualitative model space. More specifically, the mutated value *C*′ is centred around the current value *C*. This works fine for continuous functions, because the corresponding fitness landscapes are smooth and search in the neighbourhood area is likely to find better solutions. But in the qualitative model space, adjacent constraints within the same slot normally do not have the same neighbourhood relations as in the continuous search space, because these constraints are randomly ordered within the same slot.

The above facts motivate us to develop a novel mutation method suitable for the qualitative model space, and we expect that on one hand the features of the original Opt-AiNet mutation operation can be preserved to some extent, and on the other hand after mutation the new antibody is more likely to attain a better position in the search space. Based on these consideration, the following mutation operation is proposed:

(15) $$C^{\prime }=\left\{\begin{array}{cc} C & \;\mathrm{if}\;\;U(0,1)<\alpha \times N(0,1)\\ U(1,n) & \;\mathrm{otherwise} \end{array}\right)$$

In the above, *C*′, *C*, *N*(0, 1), and *α* have the same meaning as those defined in Eqs. (13), (14). *U*(0, 1) is a uniformly distributed random number with the range [0,1]. Similarly, *U*(1, *n*) stands for a uniformly distributed random number with the range [1, *n*], where *n* is the number of constraints in the current slot of the antibody. This new mutation operator first determines whether a slot should be mutated. The probability of mutating is proportional to the fitness value of the current antibody. Once the current slot is set to mutate, the mutation will follow the uniform distribution.

In this paper QML-AiNet using both the original mutation operator and the novel one will be compared in terms of their effectiveness in Section 6. For ease of description, QML-AiNet using the modified mutation method will be denoted QML-AiNet(MM) in this paper, while the one use the original mutation method is denoted QML-AiNet(OM).

### 5.5 Affinity

In Opt-AiNet the affinity is defined as the Euclidean distance between two antibodies. In QML-AiNet because we use the integer decoding strategy, and each antibody represents a qualitative model, we define the affinity between two antibodies as the “model distance” between two models which these two antibodies represent. The model distance between two models is defined as the number of different constraints in these two models.

### 5.6 The detailed steps of QML-AiNet

The steps of QML-AiNet follow the framework of opt-AiNet. First we list the parameters used by the algorithm in Table 5.

**Table 5:** Parameters in QML-AiNet.

Name	Meaning
*N*_*i*_	Number of initial antibodies in the population
*N*_*c*_	Number of clones for each antibody
*AvgFitError*	Threshold determines the stability of population
*Supp*	The suppression threshold
*d*	The percentage of new antibodies to be added into the population

The steps of the proposed QML-AiNet algorithm are given in detail as follows:

*Step 1*: Randomly generate *N*_*i*_ antibodies.
  While (stop criteria are not satisfied) iteratively execute *Step 2*–*Step 4*:
*Step 2*: Clonal selection
  • *Step 2-1*: Antibody fitness evaluation: calculate the fitness values of all antibodies according to the description in Section 5.3.
  • *Step 2-2*: Clone: Generate *N*_*c*_ clones for each antibody.
  • *Step 2-3*: Mutation: Each antibody will be mutated according to the description in Section 5.4. In particular, the original and modified mutation operators will both be tested.
  • *Step 2-4*: Fitness Evaluation: evaluate all the newly cloned antibodies. Calculate the normalised fitness value for each antibody.
  • *Step 2-5*: Selection: Select the antibody which has the biggest fitness value from each parent antibody and its clones. All the selected antibodies construct a new antibody population.
  • *Step 2-6*: Average Fitness Error Calculation: Calculate the average fitness of the new population. If the difference between the old average fitness and new average fitness is bigger than the given threshold *AvgFitError*, repeat Step 2; otherwise proceed to Step 3.
*Step 3*: Network Suppression: Each antibody interacts with others. If the affinity of two antibodies (defined in Section 5.5), is less than the suppression threshold *Supp*, the one with the smaller fitness value will be removed.
*Step 4*: Add *d* percent of the randomly generated antibodies to the population.

## 6 Experiments

In this section we evaluate QML-AiNet through a series of experiments. First, we give a brief introduction to the compartmental models, which are used as the test bed in this research. Second, we present the experimental design. Finally we report the experimental results and analysis of these results.

### 6.1 Experimental test bed

We select the compartmental models [39] as the experimental test bed (the same models were also used in [24]). The compartmental models are abstractions of many dynamic systems, and their applications have been found in many disciplines, such as pharmokinetics [40], physiology [41], epidemiology [42], and ecology [43]. Several *de facto* benchmarks in the QR community such as the single-tank, U-Tube, have their analogous compartmental models. The detailed description of these benchmarks is given by [22], and we chose the three and four-compartment models (termed models CM2_Ex3 and CM2_Ex4) as in [24] to test the performance of all algorithms.

**Fig. 4:**
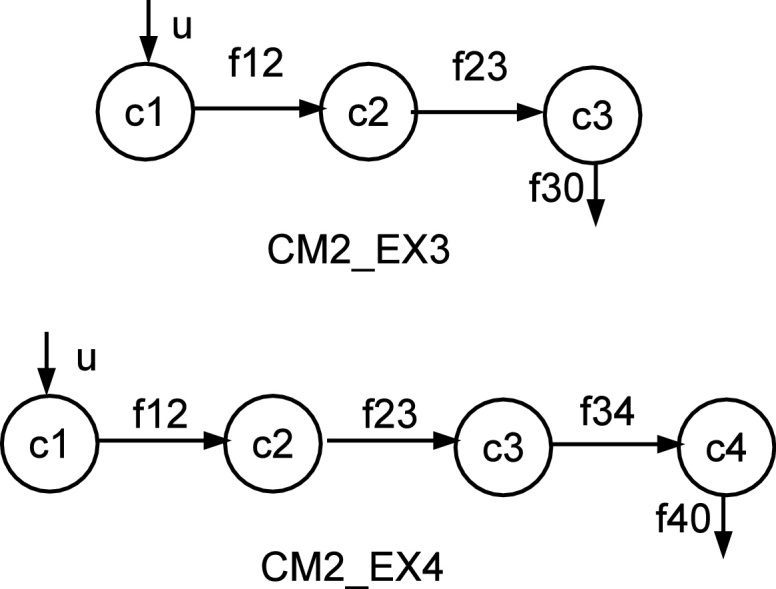
The compartmental models.

These two compartmental models are shown in Fig. 4. In this figure, *c1*, *c2*, *c3*, and *c4* stand for the concentrations in the compartments, and they are also used to “label” the compartments; *f12*, *f23*, and *f34* denote the flows from one compartment to another; *u* is the input flow; *f30* and *f40* are the output flows to the environment.

**Fig. 5:**
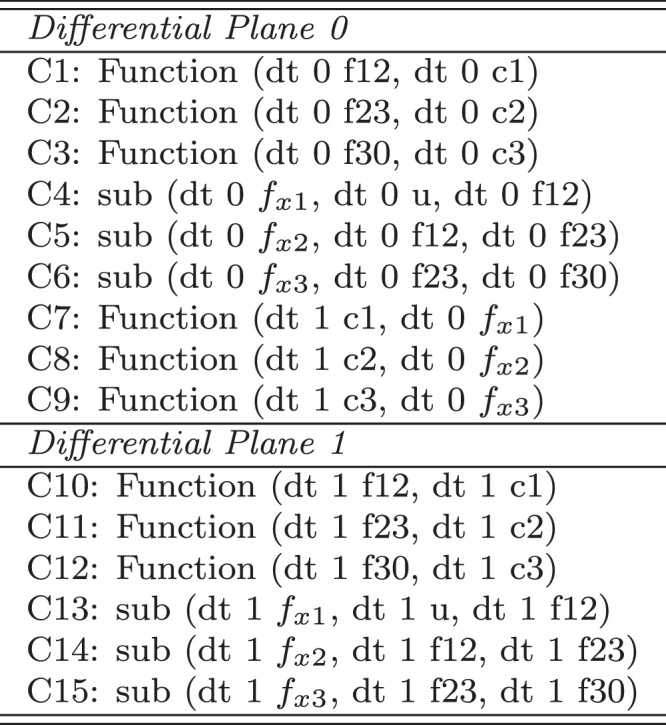
The *Morven* model for CM2_Ex3.

**Fig. 6:**
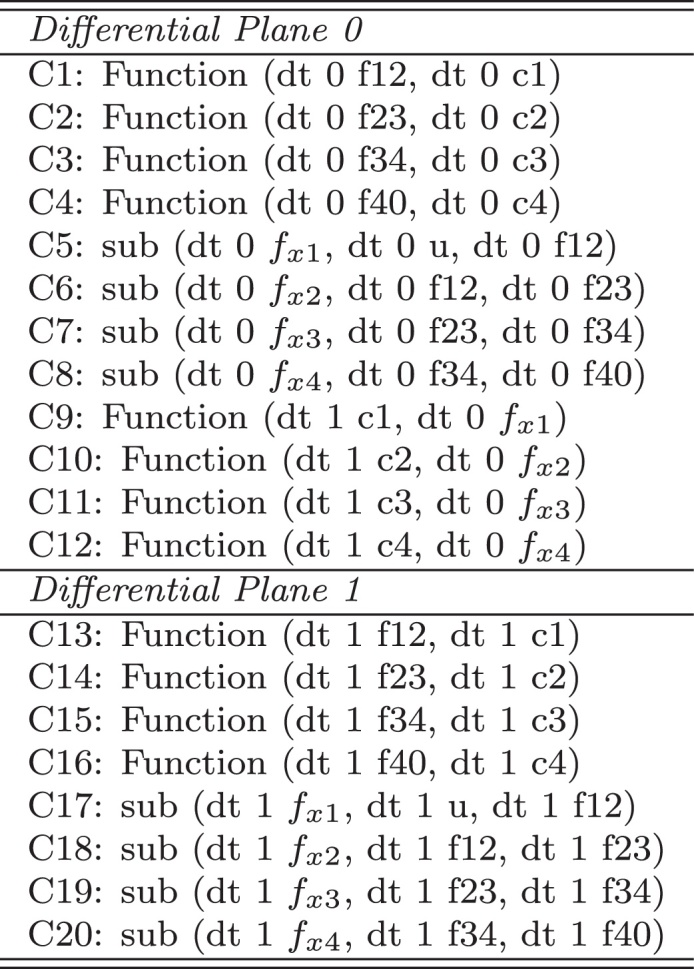
The *Morven* model for CM2_Ex4.

The QDE model of Model CM2_Ex3 used in *Morven* is given in Fig. 5. In this figure, *f*_*x*1_, *f*_*x*2_, and *f*_*x*3_ are *net flows* of compartments *c1*, *c2*, and *c3*, respectively; the *function* constraint has the same definition as those defined in Table 2. Note that in this model two differential planes are used. In addition, the *Morven* model of Model CM2_Ex4 is given in Fig. 6, and the meanings of its variables are the similar to those in CM2_Ex3.

### 6.2 Experimental design

Table 6 shows all the experiments to be performed by all algorithms. We use CM2_Ex3_E1 as an example to explain the meaning of this table: in experiment CM2_Ex3_E1 the net flow *f*_*x*3_ is a hidden variable which cannot be measured. After simulating this model in JMorven, we obtained 68 qualitative states (described in Section 2.2), and the size of the search space is 6.95 × 10^8^, which is calculated according to Eq. (9).

**Table 6:** Experiment configuration.

Experiment ID	Hidden variables	Num. of states	Search space
CM2_Ex3_E1	*f*_*x*3_	68	6.95×10^8^
CM2_Ex3_E2	*f*_*x*2_, *f*_*x*3_	48	4.81×10^1^0
CM2_Ex3_E3	*f*_*x*1_, *f*_*x*2_, *f*_*x*3_	48	6.31×10^1^1
CM2_Ex4_E2	*f*_*x*4_	340	4.22×10^1^2
CM2_Ex4_E4	*f*_*x*1_, *f*_*x*2_, *f*_*x*3_, *f*_*x*4_	164	4.74×10^1^7

The parameter values used in QML-CLONALG are: the population size is 100 for CM2_Ex3_E1 and 1000 for others; the clone size is 10; the hyper-mutation probability is 0.9; the survival time for all antibodies is 1000 generations. The parameter values used in both QML-AiNet(OM), the one with the original mutation operator, and QML-AiNet(MM), the one with the modified mutation operator, are as follows: the number of initial cells *N*_*i*_ is 20; the clone size *N*_*c*_ is 10; *AvgFitError* is 0.001; the suppression threshold *supp* is 6; *d* is 0.4.

The values of these parameters are determined by the complexity and features of the search space, and also based on performance considerations. In addition, we use the totally random algorithm as a bottom line for these three algorithms. In all experiments, complete qualitative data were used, and the stop criterion is that a well-posed model that can cover all the given data is found. All the experiments are performed on a computer cluster with 43 compute nodes, each of which has 16 Intel XEON E5520 (2.26 GHz) CPUs and 11.7 GB RAM.

### 6.3 Experimental results and analysis

For the first four experiments listed in Table 6, each of the four algorithms is run for ten trials and the best and average running time is recorded. For the last experiment CM2_Ex4_E4, due to the complexity of the problem and the limited computational resources provided by the cluster, each of the four algorithms is run for five trials, and the maximum time allowed for each trial running on the cluster is around 75 days.

To better analyse the experimental results, we will use both parametric and non-parametric statistical methods [44]. Parametric statistical methods make assumptions about the distribution of the data (e.g., normal distribution), and they have been intensively used for evaluating the performance of soft computing algorithms. Non-parametric methods, also called distribution-free methods, do not assume the distribution of the data. Recently non-parametric methods have also been used for performance comparison of soft computing algorithms [45], and they have been proven to be effective approaches. Among many non-parametric methods the Wilcoxon test [46], [47] are particularly suitable for comparing soft computing algorithms [45].

#### 6.3.1 Parametric statistical test

The experimental results are shown in Table 7, Table 8, respectively.

**Table 7:** Experimental results: best running time (ms).

Experiment ID	Random algorithm	QML-CLONALG	QML-AiNet(OM)	QML-AiNet(MM)
CM2_Ex3_E1	259,003	4,516	3,216	892
CM2_Ex3_E2	709,127	247,067	434,236	6,095
CM2_Ex3_E3	3,898,710	20,211	1,507,146	21,678
CM2_Ex4_E2	107,570,008	19,517,666	49,291,177	81,808
CM2_Ex4_E4	>6, 585, 900, 000^a^	>6, 585, 900, 000^a^	>6, 585, 900, 000^a^	6,669,020

a No target model was found in 6,585,900,000 ms (≈ 75 days).

**Table 8:** Experimental results: average running time (ms).

Experiment ID	Random algorithm	QML-CLONALG	QML-AiNet(OM)	QML-AiNet(MM)
CM2_Ex3_E1	689,662	50,082	144,958	9,096
CM2_Ex3_E2	48,334,152	1,362,385	4,888,222	198,390
CM2_Ex3_E3	106,941,796	14,219,243	12,716,194	482,396
CM2_Ex4_E2	1,822,075,689	184,163,947	188,650,143	1,175,195
CM2_Ex4_E4	>6, 585, 900, 000^a^	>6, 585, 900, 000^a^	>6, 585, 900, 000^a^	2,157,371,469

a No target model was found in 6,585,900,000 ms (≈ 75 days).

**Fig. 7:**
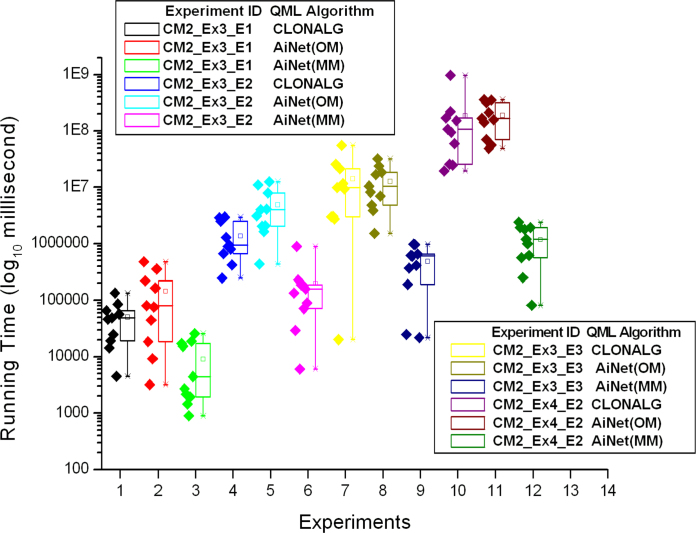
Ten trials of the first four experiments with CLONALG, AiNet (OM), and AiNet(MM).

**Fig. 8:**
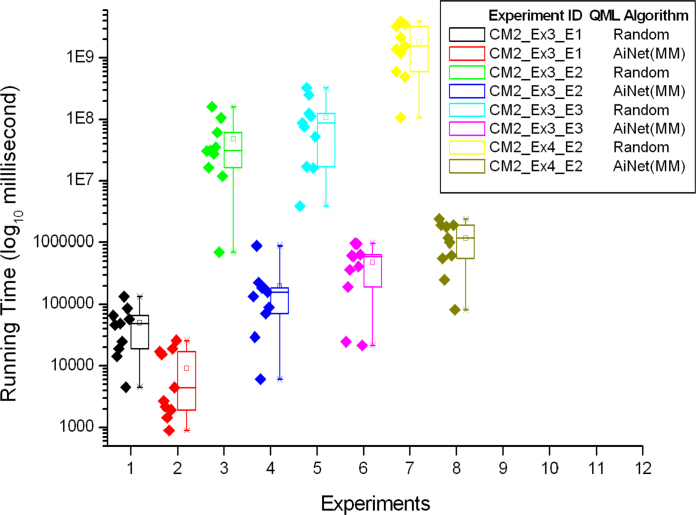
Ten trials of the first four experiments with totally random algorithm and AiNet(MM).

The details of the ten trials for the first four experiments in Table 6 are shown in Fig. 7, Fig. 8. More specifically, Fig. 7 shows the performance comparison among QML-CLONALG, QML-AiNet (OM), and QML-AiNet (MM), and Fig. 8 shows the comparison between the totally random algorithm and QML-AiNet (MM), which clearly demonstrates the scalability of QML-AiNet (MM). In these two figures, the values on the vertical axis are the running time of the algorithms, and the vertical axis is on a base-10 logarithmic scale and its unit of time is milliseconds. The diamonds on the left side of each Box-and-Whisker plot represent the running time of individual trials.

**Fig. 9:**
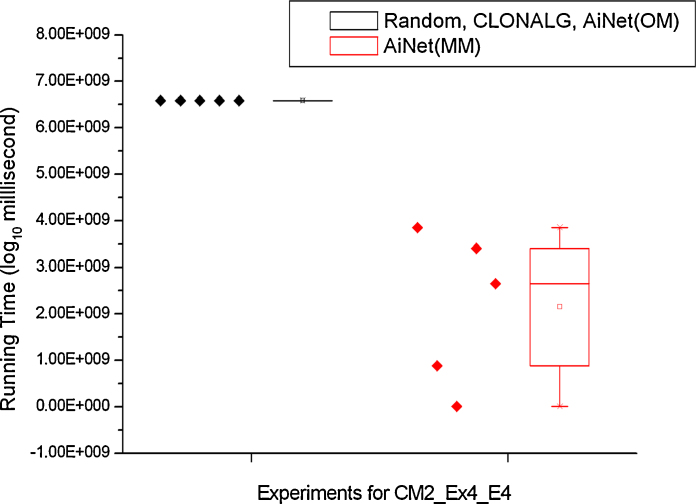
Five trials of experiment CM2_Ex4_E4.

Similarly, the details of the five trials for experiments CM2_Ex4_E4 using QML-AiNet(MM) and other three algorithms (Random, QML-CLONALG, and QML-AiNet(OM)) are shown in Fig. 9. In this figure, because apart from QML-AiNet(MM) all other three algorithm could not find the right model within the maximum allowed time (≈75 days), which is determined by the limited computational resources provided by the cluster, all five trials for these three algorithms had to stop at the same time.

From the results presented in Table 7, Table 8, as well as Fig. 7, Fig. 8, Fig. 9, one can see, that compared with the totally random algorithm, all three immune-inspired approaches improved the scalability of QML. A comparison between QML-AiNet(OM) and QML-CLONALG told us that the performance of QML-AiNet(OM) is comparable to that of QML-CLONALG. In particular, QML-AiNet(OM) outperformed QML-CLONALG in one of the first four experiments (Experiment CM2_Ex3_E3).

More importantly, QML-AiNet(MM) is much more efficient than both QML-AiNet(OM) and QML-CLONALG, and the bigger the size of the search space, the better the performance QML-AiNet(MM) can achieve compared with the other two algorithms. In terms of the average running time, QML-AiNet(MM) is two orders of magnitude faster than QML-CLONALG and QML-AiNet(OM), and three orders of magnitude faster than the totally random algorithm in Experiment CM2_EX4_E2. For the most complicated Experiment CM2_Ex4_E4, QML-AiNet(MM) is at least three orders of magnitude faster than the other two immune algorithms in terms of the best running time. All these results demonstrated the scalability of QML-AiNet(MM) with the increase of the size of search space.

#### 6.3.2 Non-parametric statistical test

To further verify and confirm our conclusion drawn from the parametric statistical test in Section 6.3.1, we use the Wilcoxon test [46], [47], a non-parametric statistical method, for comparing the performance of QML-CLONALG, OML-AiNet (OM), and QML-AiNet (MM). Table 9 shows the Wilcoxon test results based on the ten trials of each algorithm on each experiment, and the reader is referred to [45] for the details of using the Wilcoxon test for comparing different metaheuristic algorithms.

**Table 9:** Wilcoxon test results.

Experiment ID	QML-AiNet (MM) vs QML-AiNet (OM)	QML-AiNet (MM) vs QML-CLONALG	QML-AiNet (OM) vs QML-CLONALG
CM2_Ex3_E1	0.00512/0 +	0.00932/2 +	0.09296/11?
CM2_Ex3_E2	0.00694/1 +	0.0164/4 +	0.0164/4 −
CM2_Ex3_E3	0.00512/0 +	0.00694/1 +	0.88076/26 ?
CM2_Ex4_E2	0.00512/0 +	0.00512/0 +	0.20408/15 ?

In the Wilcoxon test presented in Table 9, we specify the significance level *p* to be 0.05 and use the two-tailed hypothesis because these are the most commonly used settings. As for each algorithm on each experiment we have ten trials (which means the sample size *N* = 10), we will calculate both *p*-value and *W*-value to compare the performance of each pair of the algorithms. If the *p*-value is less than the significance level (*p* = 0.05), we will say that there is a significant difference between the performance of the two algorithms. In addition, according to Table B.3 listed in [47], the critical value of *W* for *N* = 10 at *p* ≤ 0.05 is 8. This means a *W*-value less than the critical value 8 will indicate that the performance of the two algorithms is significantly different.

Once we know that the performance of two algorithms is significantly different, to further determine which algorithm performs better we will look at the sum of positive difference ranks (∑*R*_+_) and the sum of negative difference ranks (∑*R*_−_) [47], that is, if ∑*R*_+_ is less than ∑*R*_−_, we say the first algorithm outperforms the second one (because the first algorithm takes less time to complete than the second one).

For each cell in Table 9, the number before the slash is the *p*-value and the number after the slash is the *W*-value. A “+” sign means that the performance of the two algorithms is significantly different and the first algorithm outperforms the second one (∑*R*_+_ < ∑*R*_−_). Similarly, a “−” sign means ∑*R*_+_ > ∑*R*_−_ and indicates that the second algorithm outperforms the first one. If there is no significant difference between the two algorithms, we will use a “?” sign. For instance, the cell in the second row and second column of Table 9 has the data “0.00512/0 +”, which means that the *p*-value is 0.00512 and the *W*-value is 0. As the *p*-value is less than the significance level *p*=0.05, the *W*-value is less than the critical value 8, and “+” means ∑*R*_+_ < ∑*R*_−_, we can say that QML-AiNet (MM) outperforms QML-AiNet (OM) at *p*=0.05.

From Table 9 we can see that results obtained from the Wilcoxon test confirm the conclusions drawn from Section 6.3.1, that is, QML-AiNet (MM) outperforms both QML-AiNet (OM) and QML-CLONALG in all four experiments. Especially in Experiment CM2_Ex4_E2, the most complicated one, we obtained very small *p*-value and *W*-value when comparing QML-AiNet (MM) with the other two algorithms.

Interestingly, the Wilcoxon test also reveals new information when comparing QML-AiNet (OM) and QML-CLONALG. From Table 9 we see that there is no significant difference between these two algorithms on three of the four experiments. In addition, in Experiment CM2_Ex3_E2 QML-CLONALG outperforms QML-AiNet (OM). Comparing these results with those in Table 8, we can be more confident to say that the performance of QML-AiNet (OM) and QML-CLONALG is comparable. For instance, in Experiment CM2_Ex3_E1 the average performance of QML-AiNet (OM) is much lower than that of QML-AiNet (OM). However, the Wilcoxon test suggests that the performance difference is not significant. From Table 8 we see that the average performance of QML-AiNet (OM) is higher than that of QML-CLONALG in Experiment CM2_Ex3_E3 and lower than that of QML-CLONALG in Experiment CM2_Ex4_E2, but the Wilcoxon test again suggests that such difference is not significant.

#### 6.3.3 Remarks

In this section we conducted a series of experiments and used both parametric and non-parametric methods to analyse the experimental results. Experimental results and analysis upon them confirm that QML-AiNet (MM) is a very effective and efficient qualitative model learning algorithm.

The success of QML-AiNet(MM) is ascribed to its combination of Opt-AiNet style search and the mutation operator adapted to the discrete qualitative model space. The Opt-AiNet applied to QML is naturally suitable for dealing with multi-modal search space. From experimental results we observed that a straightforward adaptation of Opt-AiNet to QML, resulting in the QML-AiNet(OM) system, could improve the scalability of QML, and the performance of QML-AiNet(OM) was comparable to that of QML-CLONALG. Furthermore, after considering the features of the qualitative model space, we modified the mutation operator accordingly, which significantly improved the learning performance, resulting a more efficient system QML-AiNet(MM). This clearly shows the advantages of Opt-AiNet as a search strategy applied to QML. Finally, the parameter values of QML-AiNet(OM) and QML-AiNet(MM) are the same when performing all experiments. This indicates that it is solely the mutation operation that contributes to the improvement.

## 7 Conclusions and future work

In this paper, based on previous work about immune inspired approaches to QML, we continue to investigate the immune network approach to QML. Through this research we know that a straightforward adaptation of Opt-AiNet to QML, resulting in the proposed system QML-AiNet, could easily fulfil the goal of improving the scalability of QML, and the performance of such implementation is comparable to the previous QML system using CLONALG. More importantly, it is also indicated by experimental results that by further employing the problem-dependent mutation strategy, the performance of QML-AiNet could be even further improved. This demonstrates the potential of immune network approaches to QML.

Finally, we point out that QML is a discrete optimisation problem because of its discrete model space. So the development of QML-AiNet inspires us to explore the potential of the Opt-AiNet approach to general discrete optimisation problems. In particular, we would like to further investigate how to design suitable mutation methods to make the Opt-AiNet approach adapt to typical discrete optimisation problems, such as the knapsack, set covering, and travelling salesman problems.
